# Comparison of single coracoclavicular suture fixation and hook plate for the treatment of acute unstable distal clavicle fractures

**DOI:** 10.1186/1749-799X-9-42

**Published:** 2014-05-29

**Authors:** Chun-Yu Chen, Shan-Wei Yang, Kuan-Yu Lin, Kai-Cheng Lin, Yih-Wen Tarng, Jenn-Huei Renn, Chia-Hsin Lai

**Affiliations:** 1 Department of Orthopedics, Kaohsiung Veterans General Hospital, 386 Ta-Chung 1st Road, Kaohsiung 81346, Taiwan; 2 Department of Physical Therapy, TzuHui Institute Technology, Pingtung, Pingtung County 926, Taiwan

**Keywords:** Distal clavicle fractures, Hook plate, Coracoclavicular fixation

## Abstract

**Background:**

Surgical managements are recommended for unstable distal clavicle fractures because of a high incidence of nonunion. A variety of methods have been previously reported, but there is no current consensus regarding which method is the most suitable.

**Methods:**

Between December 2004 and August 2010, we treated 68 patients with Neer type IIB distal clavicle fractures using single coracoclavicular suture fixation with Mersilene tape (M group) or clavicular hook plate (H group). Sixty-eight patients were followed at least 24 months (mean, 37.9 months). We retrospectively compared the functional outcome, parameters, and perioperative course of the two treatments. Statistical analysis was performed with independent sample *t* test and chi-square test.

**Results:**

The M group presented significantly less operation time (*P* = 0.005) and intra-operative blood loss (*P* = 0.010) than the H group. The mean University of California at Los Angeles (UCLA) shoulder rating scale, Oxford shoulder score, VAS scale, and satisfaction score revealed no significant difference between the M group and the H group. The M group had better range of motion in the operated shoulder during forward flexion and abduction at 3 and 6 months postoperatively. However, the range of motion at 1 and 2 years after operation revealed almost the same results. Two acromial osteolysis and one acromial fracture were noted in the H group and one superficial wound infection and one frozen shoulder in the M group during follow-up. Finally, there was no significant difference in the complication rate between the two groups, and all fractures achieved union clinically at final follow-up.

**Conclusions:**

Both single coracoclavicular suture fixation and clavicular hook plate offered effective treatment in acute unstable distal clavicle fractures. However, single coracoclavicular suture fixation with Mersilene tape provided early recovery of shoulder motion and avoided further morbidity of the acromion.

## Background

Fracture of the distal clavicle accounts for approximately 21% of all clavicle fractures [1]. Distal clavicle fractures are typically attributable to a fall on an outstretched hand or a direct blow to the point of the shoulder. Neer [2,3] classified distal clavicle fractures into three types according to the relationship of the fracture line to the coracoclavicular (CC) ligaments and the acromioclavicular (AC) joint. Type I fractures are lateral to the CC ligaments with typically minimal displacement. Type III fractures involve the articular surface of the AC joints with intact CC ligaments. They are relatively stable because the proximal fragment is stabilized by the CC ligaments, and surgical intervention is usually not required. Type II fractures are subcategorized into type IIA, in which the fractures lie medial to the CC ligaments, and type IIB, in which the fractures lie more laterally with disruption of the CC ligaments from the proximal fragment.

In type IIB fractures which are unstable fractures, the weight of the arm moves the distal fragment downward and the force of the sternocleidomastoid muscle moves the proximal fragment upward; these forces cause wide displacement and difficulty in maintaining reduction with conservative treatment. Several studies [4-7] have observed high nonunion rate with conservative treatment in these unstable distal clavicle fractures, and surgery is recommended. The unstable fractures seem to represent more challenges because of the loss of attachment of the CC ligaments to the clavicle. A variety of methods of surgical fixation to treat these unstable fractures have been previously reported, including Kirschner wires [3], Knowles pins [8,9], tension band fixation [10,11], CC fixation with sutures [12] or screws [13,14], and plate fixation [15-18]. However, there is no current consensus regarding which method is the most suitable.

The clavicular hook plate is one popular surgical method, which provides rigid fixation and good bony union rates [15,16]. However, only few report on the comparison of the clinical results of the clavicular hook plate with other fixation methods [19]. Another simple surgical method for acute unstable distal clavicle fractures is single CC suture fixation with Mersilene tape; the published case series [12] demonstrated high union rate and good functional outcome. Both surgical methods could provide good functional results for patients in our clinical experience. However, there was no report of comparison between them in the literature reviews.

The purpose of this study was to retrospectively evaluate the clinical results and efficacy comparison of single CC suture fixation with Mersilene tape versus clavicular hook plating for the treatment of acute unstable distal clavicle fractures. We hypothesized that single CC suture fixation with Mersilene tape would provide a better outcome to recovery of shoulder motion and avoid acromion morbidity and fewer complications than that with the clavicular hook plate.

## Methods

### Study population

**Figure 1:**
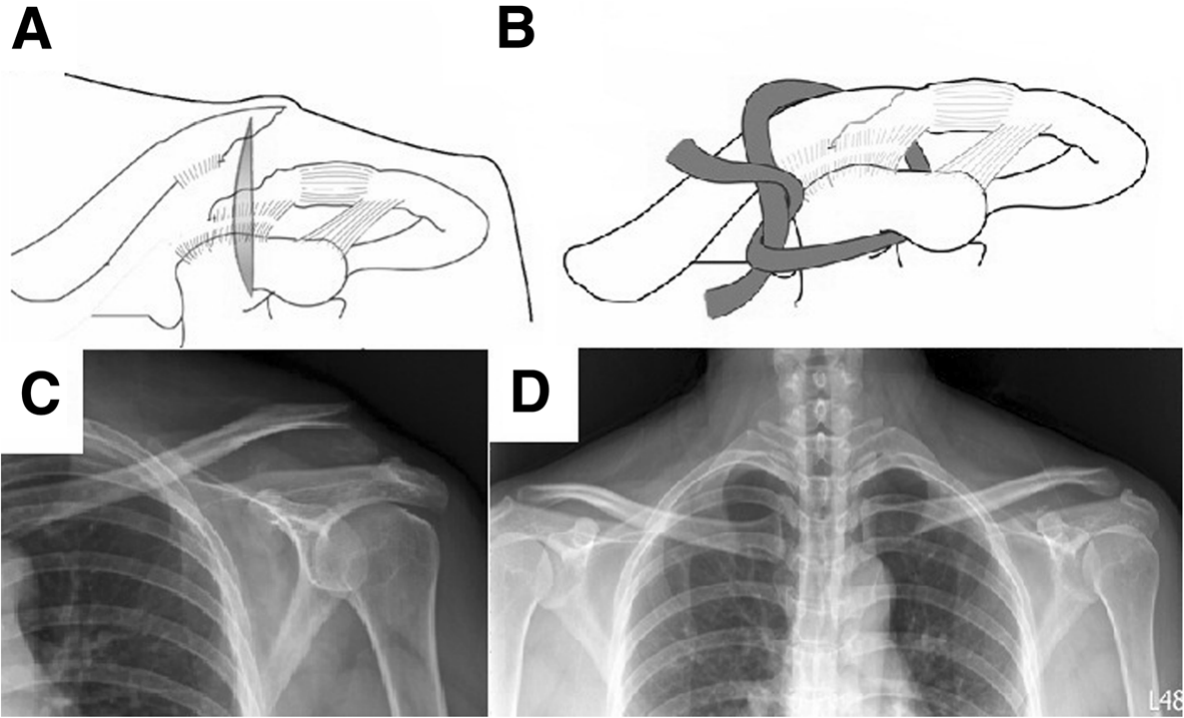
**Stabilization by single CC suture fixation with Mersilene tape.** Surgical procedure: **(A)** A longitudinal incision was made above the coracoid process. **(B)** Mersilene tape was passed around the base of the coracoid process and the medial clavicular fragment and was tied to maintain reduction. Radiography of the injured clavicle: **(C)** Preoperation. **(D)** Postfixation.

**Figure 2:**
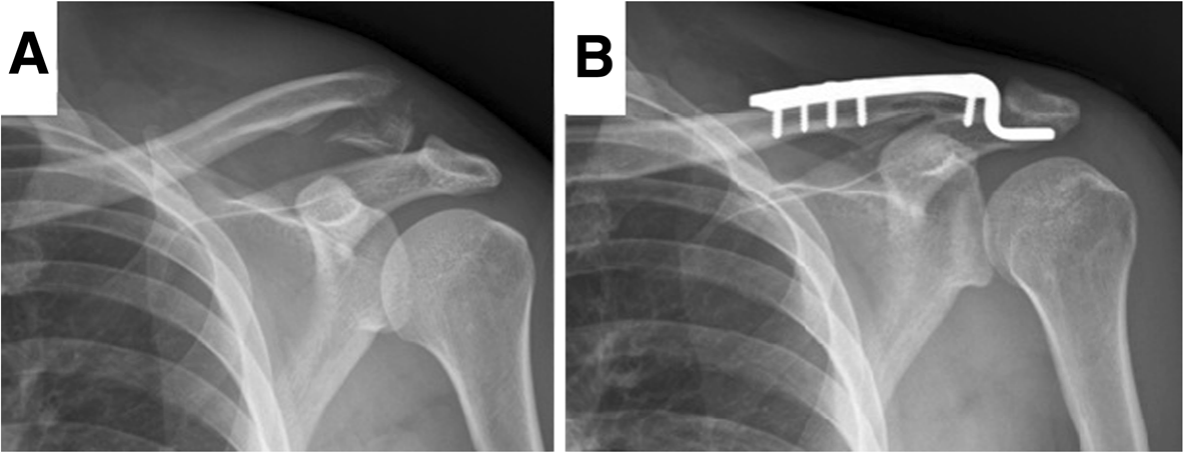
**Stabilization by hook plate. (A)** Preoperative radiography. **(B)** Postfixation radiography.

Between December 2004 and August 2010, 94 consecutive patients with Neer type IIB distal clavicular fractures were surgically treated. We selected the patients with the following criteria: (1) adults with acute, closed, and unilateral fractures; (2) fixation by single CC suture fixation with Mersilene tape or clavicular hook plating; (3) normal shoulder function before injury; (4) without associated injuries; (5) regular follow-up more than 24 months postoperatively. Sixty-eight patients fitting the criteria were included in this study. This retrospective study was approved by the institutional review board of Kaohsiung General Veterans Hospital. The patients were divided into two groups according to the surgical methods. Forty fractures were stabilized by single CC suture fixation with Mersilene tape (M group) (Figure 1). Twenty-eight were treated with clavicular hook plating (H group) (Figure 2). The demographics and injury mechanisms related to the two groups are shown in Table 1.

**Table 1:** Patient demographics and clinical characteristics

	**M group**	**H group**	***P*** **value**
Number of patients	40	28	
Gender, M/F	28/12	16/12	0.311^a^
Age (years)	43.2 (18–75)	48.3 (28–78)	0.201^b^
Trauma mechanism, V/F	24/16	20/8	0.441^a^
Time from injury to surgery (days)	1.6 (0–4)	1.8 (0–5)	0.791^b^
Follow-up period (months)	38.2 (24–64)	37.4 (24–68)	0.460^b^

Data are expressed as mean (range) unless stated otherwise; *M* single coracoclavicular suture fixation with Mersilene tape, *H* AO hook plating, *V*/*F* vehicular trauma/fall from a height; ^a^chi-square test; ^b^independent sample *t* test.

### Surgical technique - M group

A longitudinal skin incision was made from the distal clavicle to the coracoid process. The anterior deltoid muscle was split from the medial segment of the clavicle to access the base of the coracoid process. A right-angle clamp was used to pass the Mersilene tape (Ethicon, Somerville, NJ, USA) through the inferior base of the coracoid process and around the medial clavicular segment. Pressing the medial fragment down until it contacted the lateral fragment in an anatomic position, the Mersilene tape was tied for CC stabilization.

### Surgical technique - H group

A linear incision parallel to the distal clavicle was made. After the fracture site was exposed and reduced, and the subacromial space was confirmed, the hook portion of the plate (Synthes-Stratec Medical, Solothurn, Switzerland) was inserted under the acromion. The clavicle portion of the plate was contoured and fixed with screws.

### Postoperative rehabilitation

Gentle pendulum exercise was encouraged postoperatively under the protection of an arm-sling for 4 weeks followed with active range of motion of the affected shoulder gradually in both groups.

### Clinical evaluations

All patients received monthly radiographs and clinical follow-up until union. Successful union was defined by obliteration of the fracture gap on plain radiographs and no tenderness or pain at the fracture site during shoulder exercise. All medical records and radiographic examinations were retrospectively reviewed to compare operation time, blood loss after operation, union time, complications, and active shoulder range of motion (ROM) at every outpatient department follow-up. Clinical recovery was evaluated at least 24 months postoperatively according to the questionnaires, including (1) the patient-completed Oxford shoulder score [20] which was stratified into satisfactory function (40–48 points), mild to moderate dysfunction (30–39 points), moderate to severe dysfunction (20–29 points), or severe dysfunction (0–19 points) and (2) the clinician-completed University of California at Los Angeles (UCLA) shoulder rating scale [21] which was stratified into good to excellent result (27–35 points) or fair to poor result (<27 points). The visual analog scale (0–10) of pain and satisfaction score (0–10) were also analyzed in detail. Three experienced orthopedic trauma surgeons (Yang SW, Lin KU, and Lin KC) treated the patients and randomly decided on the surgical method according to personal experience. However, the results of all patients were followed and reviewed by one single independent observer.

### Statistical analysis

The data were analyzed using independent sample *t* test and chi-square test to compare perioperative parameters. Significance of differences across the two groups in terms of mean scores was assessed using the Mann–Whitney *U* test. The statistical analysis was performed by using the Statistical Package for the Social Sciences (SPSS version 17.0, SPSS, Chicago, IL, USA). The chosen level of significance was *P* < 0.05.

## Results

### Demographics and perioperative parameters

No significant differences existed in gender, mean age, trauma mechanism, average time from injury to surgery, and mean follow-up period between the two groups (Table 1). The perioperative parameters and clinical results of the two groups are presented in Table 2. The M group presented significantly less operation time (*P* = 0.005) and intra-operative blood loss (*P* = 0.010) than the H group.

**Table 2:** Perioperative measurements and clinical results

	**M group**	**H group**	***P*** **value**
Operation time (min)	43.5 (30 ~ 80)	65.63 (45 ~ 87)	0.005^a^
Blood loss (ml)	22.3 (10 ~ 50)	55 (25 ~ 100)	0.010^a^
Complication rate, *n* (%)	2/40 (5)	3/28 (10.7)	0.396^b^
Union rate, *n* (%)	40/40 (100)	28/28 (100)	

Data are expressed as mean (range) unless stated otherwise; *M* single coracoclavicular suture fixation with Mersilene tape, *H* AO hook plating; ^a^independent sample *t* test; ^b^chi-square test.

### Clinical outcomes

**Figure 3:**
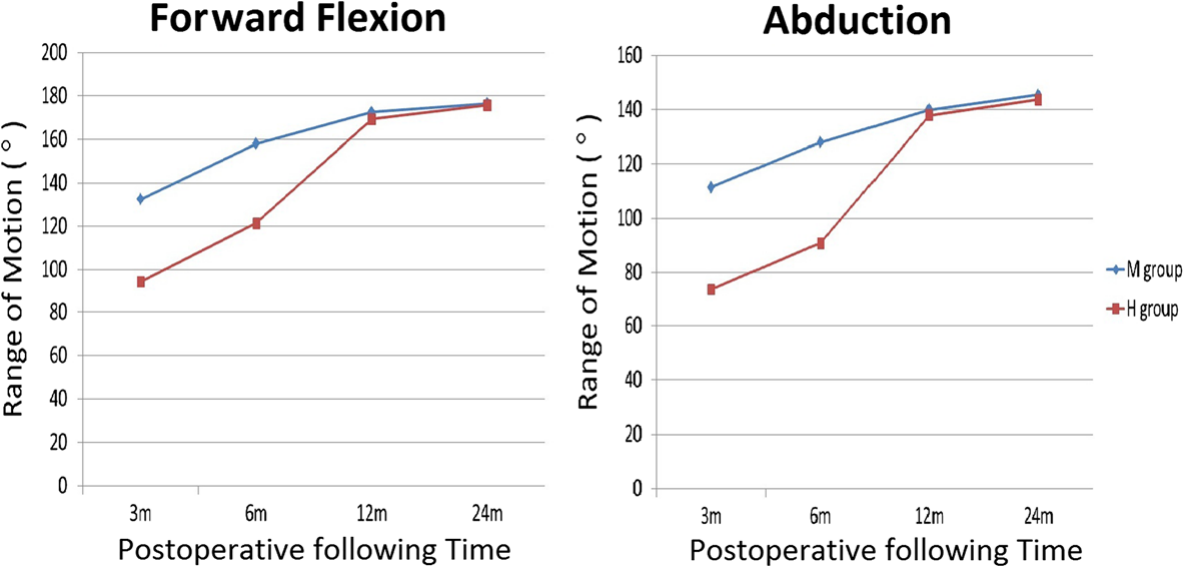
Range of motion of the operated shoulder during follow-up postoperatively.

At final follow-up, both groups had relative low average pain scores and high satisfaction scores 2 years after operation, and there was no significant difference between them. The functional results including the UCLA shoulder rating scale and the Oxford shoulder score presented good results at 2 years postoperatively in both groups without a significant difference (Table 3). The evaluation of active forward elevation and abduction demonstrated that the M group had better ROM than the H group at 3 and 6 months postoperatively. However, the ROM at 1 and 2 years after operation revealed almost the same results in both groups (Figure 3).

**Table 3:** Scoring for clinical outcome at 2 years postoperatively

	**M group**	**H group**	***P*** **value (Mann–Whitney *U *** **test)**
VAS scale	0.38 (0–2)	0.5 (0–2)	0.746
Satisfaction score	9.62 (7–10)	9.5 (8–10)	0.789
UCLA scale	33.8 (30–35)	33.1 (29–35)	0.547
Oxford score	47.2 (45–48)	46.9 (45–48)	0.786

Data are expressed as mean (range); *M* single coracoclavicular suture fixation with Mersilene tape, *H* AO hook plating.

### Complications

All the fractures achieved union clinically at final follow-up. All patients in the H group received another surgery to remove the hook plate after bony union. The mean of time to remove implants was 24 weeks (range, 20–32 weeks). One patient of the M group suffered from superficial wound infection. After surgical debridement and administration of antibiotics, the wound healed well. Another patient of the M group suffered from frozen shoulder on the operated side. The symptom resolved after adequate rehabilitation without any surgical intervention. Three patients in the H group complained about implant-related discomforts during shoulder motion. Two were acromial osteolysis, and the symptom subsided after removal of hook plates. The other one was acromial fracture which resulted from erosion of the acromion by the hook plate; the patient received removal of the implant and protection by an arm-sling for 4 weeks, and no residual symptom was noted finally. However, there was no significant difference in the complication rate between the two groups by chi-square test.

## Discussion

In the current study, we compared the results between two surgical methods including AO clavicular hook plate and single CC suture fixation with Mersilene tape. Both methods could achieve union and provide good function outcome finally. However, the H group presented more blood loss and longer operative time than the M group, because application of the hook plate required wider dissection. Single CC suture fixation only needs about 3- to 5-cm longitudinal incision above the coracoid process, a less invasive procedure in contrast to open reduction for hook plate fixation; it usually requires less operative time to finish the procedure. According to the UCLA shoulder rating scale and the Oxford shoulder score, we found that both surgical methods can have similar and good functional results 2 years after the surgery and almost present normal shoulder function. This study demonstrated that the ROM of forward flexion and abduction at each time point differed significantly between the two groups at 3 and 6 months postoperatively. ElMaraghy et al. [22] reported that the subacromial hook resulted in subacromail bursal penetration and the subacromial space is limited. Because of bursal inflammation and rotator cuff impingement, the H group had the worse ROM which may have resulted from the pain during shoulder movement. In the H group, all the patients received implant removal at the mean time of 24 months. After removal, we could find that the ROM at 12 and 24 months did not differ significantly between the two groups. Otherwise, the ROM in both groups also improved than before. That is probably the main cause why the functional results of the two groups were as good as each other at final examination. Tan et al. [19] also reported that shoulder function in the hook plate group improved markedly after plate removal.

Several studies [15,16] in recent years recommended internal fixation using the AC hook plate and the AO clavicular hook plate with the lateral hook passing below the acromion posterior to the acromioclavicular joint and the subacromial hook performing a leverage to depress and fix the medial fragment. Although the clavicular hook plate provided excellent results, this implant may cause rotator cuff injury, subacromial impingement, and acromial fracture. Furthermore, a secondary operation must be performed to remove the hook plate, because long-term fixation has a considerable risk for these complications [15,16,23]. Renger et al. [16] reported three patients (6.8%) with acromial osteolysis on radiographic analysis, and 30 patients (68%) complained about implant-related discomforts during mobilization such as pain, scraping feeling, and impingement-induced limitation of range of motion. All implant-related discomforts disappeared after removal of the hook plate.

Coracoclavicular stabilization using nonabsorbable sutures such as Ethibond (Ethicon, Somerville, NJ, USA), FiberWire (Arthrex, Naples, FL, USA), and Mersilene tape (Ethicon, Somerville, NJ, USA) were used for supplementary fixation in addition to implant in several studies [12,24,25]. The sutures passed around the coracoid process and either around or through a hole of the proximal fragment. Chen et al. [24] introduced a procedure consisting of CC reconstruction with Mersilene tape, repair of torn ligament, and wire fixation of the fracture fragments to treat the distal clavicle with a disruption of the CC ligament. Checchia et al. [25] developed an all-arthroscopic technique to perform a double CC cerclage with FiberWire suture in seven cases, and results were satisfactory in all cases. Yang et al. [12] considered that repair of torn ligament and hardware fixation were not necessary, which even increased soft tissue stripping and devascularization. They reported 28 patients with Neer type IIB fractures stabilized by single CC suture fixation with Mersilene tape without any hardware implantation or CC repair. All patients returned to work and had good to excellent functional results.

Oh et al. [7] reviewed 425 Neer type II fractures from 21 studies between January 1990 to September 2009. One hundred and five patients received coracoclavicular stabilization, and 162 patients received hook plate fixation. The complication rate was significantly higher in cases with the hook plate (40.7%) than in those with coracoclavicular stabilization (4.8%). Another meta-analysis from Stegeman et al. [26] showed that hook plate fixation had a 24-fold increased risk compared to suture anchoring. These two articles both recommended a fixation procedure with a low risk of implant-related complications. In our study, the complication rate of the hook plate was not as high as that in the above studies, and the complication rate between the two groups was not statistically different. In our opinion, the major complication of hook plate fixation was shoulder impingement in motion. So all patients in our study received secondary surgical removal to avoid further acromial morbidity and improve shoulder motion. However, we still find some acromial morbidity in the H group, including acromial osteolysis and acromial fracture.

Even though we know that the hook plate has a relatively high complication rate and CC suture fixation presented more advantages than the hook plate in the present study, various limitations still existed, including the following: (1) This is a retrospective study and not randomized; (2) the operations were not performed by a single surgeon, and the level of experience could influence the outcome; (3) this study was a small series because distal clavicle fractures were relatively rare. A larger number and randomized study may be necessary to confirm the results between these methods in the future.

## Conclusions

From this study, both the surgical methods of single CC suture fixation with Mersilene tape and AO clavicular hook plate could provide good union rate and functional results for the treatment of unstable distal clavicle fractures. However, single CC suture fixation had additional advantages including less blood loss and operative time, avoidance of acromial morbidity, nonnecessity of further surgical removal of the implant, and early recovery of shoulder ROM.

## Competing interests

The authors declare that they have no competing interests.

## Authors' contributions

C-YC and S-WY conducted the retrospective study and drafted the manuscript. K-YL and K-CL supervised the research and revised the manuscript. J-HR, C-HL, and Y-WT provided assistance for data analysis and statistical analysis. All authors read and approved the final manuscript.
